# Design of a Robust Tool for Deploying Large-Area Stretchable Sensor Networks from Microscale to Macroscale

**DOI:** 10.3390/s22134856

**Published:** 2022-06-27

**Authors:** Elliot Ransom, Xiyuan Chen, Fu-Kuo Chang

**Affiliations:** 1 Department of Aeronautics and Astronautics, Stanford University, Durand Building, 496 Lomita Mall, Stanford, CA 94305, USA; ehransom@stanford.edu (E.R.); fkchang@stanford.edu (F.-K.C.); 2 Department of Mechanical Engineering, Stanford University, Building 530, 440 Escondido Mall, Stanford, CA 94305, USA

**Keywords:** sensor network, stretchable electronics deployment, smart structure, structural health monitoring

## Abstract

An investigation was conducted to develop an effective automated tool to deploy micro-fabricated stretchable networks of distributed sensors onto the surface of large structures at macroscale to create “smart” structures with embedded distributed sensor networks. Integrating a large network of distributed sensors with structures has been a major challenge in the design of so-called smart structures or devices for cyber-physical applications where a large amount of usage data from structures or devices can be generated for artificial intelligence applications. Indeed, many “island-and-serpentine”-type distributed sensor networks, while promising, remain difficult to deploy. This study aims to enable such networks to be deployed in a safe, automated, and efficient way. To this end, a scissor-hinge controlled system was proposed as the basis for a deployment mechanism for such stretchable sensor networks (SSNs). A model based on a kinematic scissor-hinge mechanism was developed to simulate and design the proposed system to automatically stretch a micro-scaled square network with uniformly distributed sensor nodes. A prototype of an automatic scissor-hinge stretchable tool was constructed during the study with an array of four scissor-hinge mechanisms, each belt-driven by a single stepper motor. Two micro-fabricated SSNs from a 100 mm wafer were fabricated at the Stanford Nanofabrication Facility for this deployment study. The networks were designed to be able to cover an area 100 times their manufacturing size (from a 100 mm diameter wafer to a 1 m${}^{2}$ active area) once stretched. It was demonstrated that the proposed deployment tool could place sensor nodes in prescribed locations efficiently within a drastically shorter time than in current labor-intensive manual deployment methods.

## 1. Introduction

The problem of embedding multimodal sensors throughout a structure is of great interest to the engineering community, especially with regard to structural health monitoring [1,2,3,4,5,6,7,8,9]. By integrating strategically placed sensors within a structure, a variety of important data can be gathered as to that structure’s state of health [3,5,7,9,10,11]. Strain data obtained from conventional strain gauges can be used to evaluate and validate design loads in a structure and to monitor loads in situ to prevent them from exceeding these design values [3,4,9,12,13]. Piezoelectric transducers are similarly useful, allowing for the monitoring of cracks or other damage within a structure and providing impact detection for the structure during its operation [3,5,6,7,9,11]. Temperature sensors such as resistive thermal devices (RTDs) allow for temperature monitoring within a structure [13,14]. This allows for evaluation of potentially dangerous thermal loads. Multimodal sensors can also serve as a control technology, as in “fly-by-feel” UAVs [15,16,17], and can provide slip and contact detection to the surface of an object such as a robotic finger [18].

A number of approaches for integrating such multifunctional sensor networks within large-area structures have been proposed and implemented within the past decade [4,19,20,21,22,23,24,25,26,27,28,29,30,31]. In this study, the focus is on the stretchable sensor network (SSN) developed at the Stanford Structures and Composites Lab (SACL) as well as many similar technologies often described as “island-and-serpentine” networks [3,13,14,22,23,32,33,34,35,36,37,38,39,40,41]. These networks comprise an array of sensor nodes connected to each other by coiled serpentine interconnects capable of expanding via rigid body rotation, preserving their resistivity as they are opened. Such networks are a compelling solution for the integration of multifunctional sensor networks within a large-area structure or part, providing a platform that can be manufactured at microscale and deployed at macroscale in a non-parasitic, simply packaged way. In this study, the aim is to enable such networks to be deployed in a safe, automated, and efficient manner, facilitating their use in a variety of tasks including structural health monitoring, medical devices, antennas, and electrodes. This is achieved using a proposed “stretch tool” capable of stretching uniform networks with a series of scissor-hinge mechanisms. The kinematic principles of the device are explained, a prototype is developed, and two test runs are conducted to determine the appropriateness of the tool for expanding SSNs. Specifically, the device is evaluated based on its preservation of the network during stretching (i.e., no broken or tangled interconnects), the process time of the stretching process, which should be on the order of one minute, and the accuracy of the tool in deploying nodes in their design positions, characterized by the mean positional error in the positions of the final nodes from a uniform grid.

### Stretchable Sensor Networks

**Figure 1:**
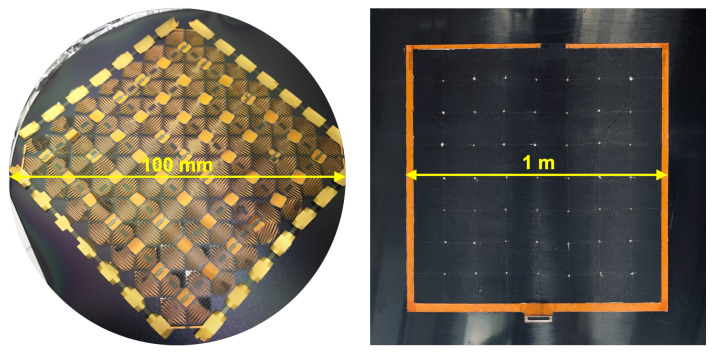
(**Left**) An SSN in its unstretched state. (**Right**) A stretched and deployed SSN on a composite panel.

As noted, SACL at Stanford University has previously developed a multifunctional network called the SSN [35,36,39]. This network supports strain gauge, RTD, and lead zirconate titanate (PZT) sensors with real-time data acquisition, and can be expanded from a 100 mm diameter wafer footprint to a square footprint of one square meter: a 100-fold area increase, shown in Figure 1. The SSN has been deployed in composite structures to monitor loads, impacts, damage, and temperature in real time.

**Figure 2:**
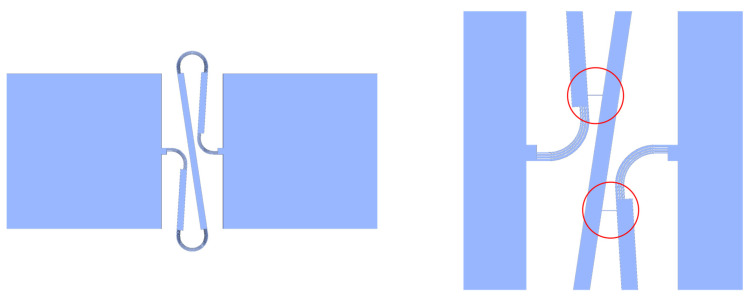
An interconnect connecting two nodes (**left**) and circled sacrifice bridges (**right**).

A critical element of the SSN is the sacrifice bridge, pictured in Figure 2. After release from the wafer, polyimide sacrifice bridges are present in the completed sensor network. The bridges prevent the interconnects from unfolding before deployment, keeping the interconnects rigid enough for the network to be manipulated and transported. During deployment, these bridges are designed to break, allowing the network to be opened to its design size.

While the SSN and its derivatives have shown the capability to perform a wide variety of structural health monitoring tasks, the actual deployment of these networks remains a thorny issue and prevents the technology from being mature in the manufacturing sense. In this paper, a method is presented for automatically deploying SSNs to cover an area with sensors resting at desired locations, closing the gap between the lab-scale manual deployment process and a mature, automated manufacturing process.

## 2. Related Works

A variety of techniques for integrating multifunctional sensor networks in large-area structures have been pursued in recent years; however, the techniques of interest in this work are “island-and-serpentine” approaches. These approaches have a history spanning just over a decade, and remain an active, if relatively small, corner of sensor network study.

One of the earlier island-and-serpentine networks is the SSN, which was developed at SACL by Lanzara et al. in 2008 and has seen continued development since that time [36,39,42,43,44,45]. A more current model of the SSN is seen in Figure 1; this network has serpentine interconnects with tessellating diamond footprints. It supports strain gauge, RTD, and PZT sensors, and has been deployed in composite structures such as plates and aircraft wings. A modified version of this network is used in this paper as the test device for the proposed deployment tool.

Since this development, other island-and-serpentine techniques have emerged. In 2012, Xu et al. innovated on the SSN design by employing “self-similar” serpentine networks to increase the expanded length of the stretched interconnects [40]. This particular network was used for a stretchable battery. The apparatus used to stretch the network, if any, is not specified.

A similar uniform square network was studied by Zhang et al. in 2013, in a study of interconnect buckling, using simple “S”-shaped interconnects [38]. The authors note in this case that they used customized uniaxial and biaxial stretchers; however, these are unfortunately not pictured or elaborated on. As the work was a treatment of the structural properties of the network, this network was not functionalized.

Additionally in 2013, researchers began to develop alternative geometries to the square island and “S”-shaped serpentine networks. Fan et al. simulated the mechanics of von Koch curve, Peano curve, Hilbert curve, Moore curve, Vicsek fractal, and Greek cross geometries, eventually fabricating a Peano curve device [41]. In this case, the networks were not expanded to a great area with any apparatus or with manual methods. The intended use of these devices are as electrodes or antennas.

In a similar vein, in 2014, Jang et al. extended the concept to different geometries [33]. Rather than using a square network, the authors of that work used triangular, honeycomb, and Kagome geometries to tailor nonlinear stress–strain responses in their networks, in part to mimic the mechanical response of the human skin. The network itself had no embedded sensors, but could potentially serve as a sensor network platform.

Another application of island-and-serpentine networks can be found in Gutbrod et al. in 2014, wherein such a device was embedded in a cardial rabbit-heart “sock” for therapeutic applications [37]. In this case, the island-and-serpentine network is not expanded, but rather conforms to a 3D geometry with aid from the serpentine structure. The network has pH sensors, and also has actuators in the form of light-emitting diodes.

In 2018, Hua et al. produced a sensor network for multifunctional sensing using a very similar serpentine interconnect design, with the goal of providing multifunctional sensing over 3D areas with arbitrary geometries. This network broadened the scope of functionality of networks of this type to include optical, humidity, and magnetic field sensing in addition to the sensor modalities supported by the SSN. This network is shown being stretched over a frame in [13], as well as being pulled apart by linear fixtures. The deployment method itself, however, is not elaborated on.

In 2018, Wang et al. developed an island-and-serpentine network for a similar application to the SSN, i.e., to perform structural health monitoring of aircraft wings [3]. This design supports PZTs for this purpose, and innovated on the SSN by introducing a fractal geometry in the serpentine interconnect structure to increase the expanded length of the interconnect. To deploy this structure, successive nodes are fixed in place while the remaining nodes are stretched; this process is performed manually and in two dimensions without the use of specialized equipment.

In Table 1 and Table 2, the works above are categorized by their geometries, deployment methods, sensor types, and applications. Recent progress in this field is also reviewed in [46,47].

While these works show promise across domains as diverse as structural health monitoring, medical devices, and multimodal sensing, most of these works performed the actual network expansion in an unspecified way, save for Fan et al., who claimed the use of specialized fixtures. Additionally, some networks are simply left unstretched, using their serpentine interconnects chiefly as a means for conformation to an arbitrary surface.

Indeed, several of the aforementioned network architectures would benefit from a means of automated deployment. In this work, the aim is to enable these promising architectures to mature from laboratory-scale deployments and prototypes to platforms capable of being deployed in a safe, convenient way.

## 3. Problem Formulation

Given a micro-fabricated stretchable network of sensor nodes fabricated at a 100 mm wafer size, as shown in Figure 1, it is desired to develop a tool to open the network from its edges to stretch that network to its intended size and then deploy it onto the surface of a plate with a 1 m${}^{2}$ active area. Until this writing, such networks have been deployed manually by researchers and technicians [14,35,36,39,48], with the exception of one other deployment performed at SACL [16]. All network nodes are individually attached to the work surface for later integration with the target structure. A series of challenges are encountered during the network stretching process. The first class of challenge is the inherent inefficiency of the process; to proceed through the entire process, the technician must manually attach, detach, and reattach each perimeter sensor node to the fixtures and work surface, making the process laborious and time-consuming. A technician pursuing this method can expect to spend on the order of hours to complete the process for an eight-by-eight square network, even if performing the process perfectly.

The second class of problem with the process concerns potential damage to the network. The serpentines on the SSN have width on the order of a human hair, with the correspondingly low structural strength and bending rigidity that this implies. Additionally, the geometry of the serpentines is prone to tangling, with hairpins in a given serpentine being likely to latch onto their neighbors. As a result, technicians run the risk of snapping interconnects with manually controlled movements, and even a careful technician may allow an interconnect to tangle with its neighbor, necessitating another potentially damaging manual process to untangle it. In many cases, a snapped interconnect can mean the disconnection of one or more sensors, and in all cases, a snapped interconnect will displace the remaining sensors, which are held in static equilibrium by tension between the interconnects.

Therefore, the objective is to develop a deployment tool that can stretch the micro-fabricated sensor networks reliably, effectively, and in the shortest time with minimum labor involvement. In summary, it is desired that (a) the perimeter nodes expand uniformly; (b) these perimeter nodes are directly controlled to follow this uniform expansion; (c) control resolution is sufficiently high such that network expansion is controlled and the risk of broken interconnects is reduced.

In order to achieve the desired objectives, development of a mechanical device or “stretch tool” comprising four scissor-hinge mechanisms driven by stepper motors is proposed.

It must first be considered how the network should behave during its expansion, then a tool should be produced that replicates this behavior. Consider the desired behavior; unlike in previous methods, which comprise two separate expansions in two different directions [48], a controlled expansion in both directions at the same time is considered. In previous methods, when the network is expanded in one direction, the edge nodes are fixed to linear fixtures which are constrained to move along a certain path; the individual tensions applied to the network interconnects are not controlled, but rather the edge nodes are positionally controlled. This philosophy is partially effective for a 1D stretch; in the new method proposed here, this simple process is extended to achieve positional control of edge nodes in two dimensions simultaneously.

The desired behavior can be visualized as follows. Initially, the nodes at the perimeter of the network are positioned in the manufacturing footprint which has a given aspect ratio. It is desired that during the expansion, these perimeter nodes expand uniformly; i.e., each perimeter node is equally spaced from its neighbors, and the perimeter always maintains the same aspect ratio during stretching. In light of this, the perimeter nodes must be positionally constrained to the tool while stretching.

The chief problem that must be addressed here is the fragility of the sensor network interconnects. Individual interconnects are very delicate, with cross-sectional area on the order of a human hair. As such, these interconnects will break easily if excess tension is applied. The tool must provide enough tension to expand these interconnects, breaking the sacrifice bridges without damaging the interconnects themselves. The minimum distance that the tool can be moved during a single control signal thus becomes very important; if this value is too large, the network interconnects may experience too much load and thus snap.

### 3.1. Scissor-Hinge Mechanisms

**Figure 3:**
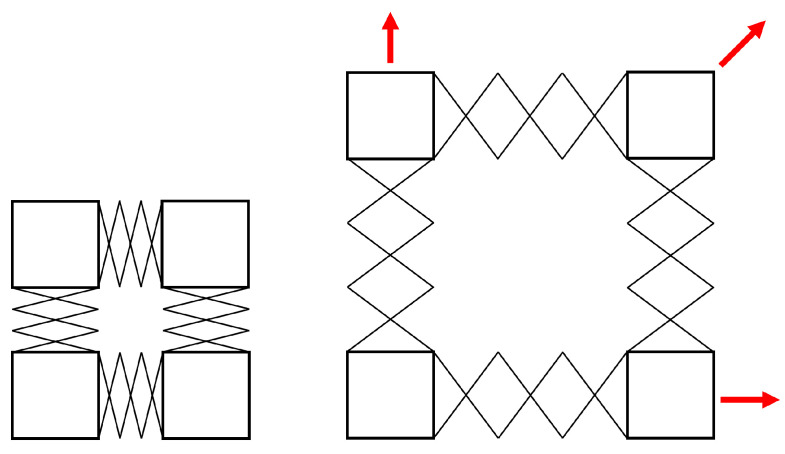
A square formation of scissor-hinge mechanisms expands as desired, with the bottom left block remaining stationary. Red arrows indicate the movement paths of the remaining blocks.

A mechanical assembly pursuant to the requirements in Section 3 is necessary. A useful mechanism for this assembly is the scissor-hinge mechanism: such a mechanism permits fixed points on the mechanism to expand uniformly. In fact, if two parallel pairs of scissor hinge mechanisms are arranged in a square formation, the desired expansion behavior can be produced: a slow, controlled expansion. Figure 3 illustrates this concept.

**Figure 4:**
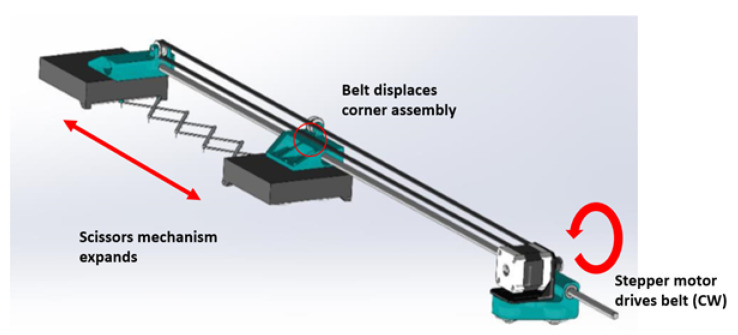
A quarter-view of the stretch tool detailing the movement of one scissor-hinge mechanism.

The expansion of the scissor-hinge mechanisms must be physically controlled. A stepper motor with a timing belt can be used to expand and contract the network. These motors can be mounted on blocks at the corners of the complete tool. A quarter-view of the assembly is shown in Figure 4.

During expansion:

1. An optical encoder is adjusted to control the stepper motor speed.
2. This signal is passed to a microcontroller, which passes motor commands through the stepper motor driver.
3. The stepper motor turns, moving the belt.
4. The belt, fixed to the block, moves the block in the expansion direction.
5. The scissor-hinge, constrained to the blocks, opens as it is pulled.

**Figure 5:**
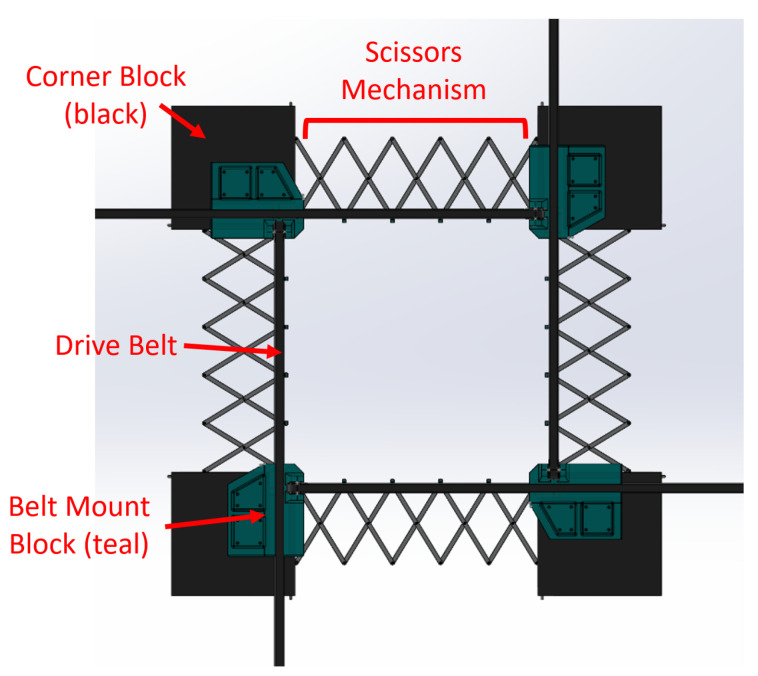
A drawing of the completed stretch tool is shown from above. Scissor-hinge links are 90 mm in length.

By assembling four such mechanisms together, the entire tool can be constructed as shown in Figure 5.

**Figure 6:**
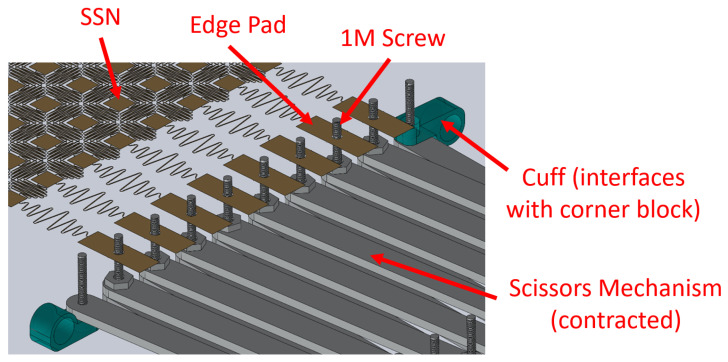
The interface between the scissor-hinge and the perimeter nodes is shown. Note the 1 M screws protruding through the through-holes in the edge nodes.

To achieve the desired expansion, each perimeter node can be fixed to an interior hinge of one of the scissor-hinge assemblies. These hinges will then expand in the desired way. In order to fix the nodes to the scissor hinges, the scissor hinges are designed with a protruding 1 M screw, and corresponding 1 mm through-holes are patterned in the perimeter nodes. These nodes can be slipped over the protruding screws to provide a mechanical connection during stretching, as shown in Figure 6.

### 3.2. Stretch Tool Design and Prototype

In this section, the design of the stretch tool is detailed, as well as the assembly of the prototype used in validation experiments.

#### 3.2.1. Important Design Parameters

After formulating this general concept, the tool must be designed to stretch a specific network. The most important considerations are movement resolution (which must be sufficiently high to permit a slow expansion), and compatibility (the scissor hinges must reach the initial and final network configurations during their movement). These considerations will later be expounded upon in detail, but in summary, the design parameters correspond to the requirements as in Table 3.

#### 3.2.2. Movement Resolution

In order to characterize the minimum amount the scissor hinges can move, the kinematics of the scissor-hinge expansion must be considered. The minimum amount the scissor hinges can move is directly related to the pitch of the belt and gear assembly; these quantities must be chosen to produce sufficiently high-resolution movement.

**Figure 7:**
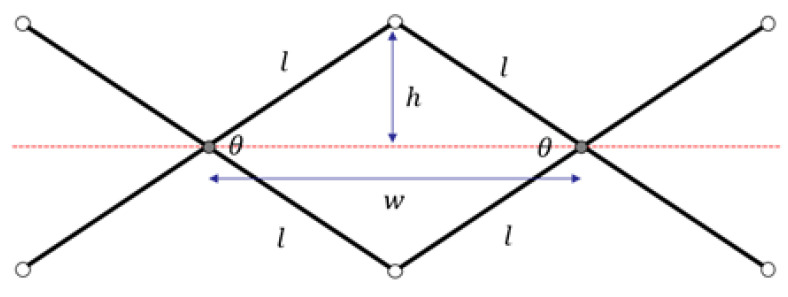
An elementary scissor-hinge unit is shown.

Scissor-hinge mechanisms are built up of scissor-hinge units. A translational deployable scissor-hinge unit consists of two straight links connected to each other at intermediate points. Scissor-hinge units of this type have well-defined kinematics. The units in the proposed tool are the simplest kind: all links are of equal length *l* and interior angle θ. Distances *w* and *h* are also defined in Figure 7.

Simple geometry can be invoked to reach the relationships in Equations (1) and (2).

(1) w=2lcos(θ/2),

(2) h=lsin(θ/2),

where *w* is the distance between intersection points and *h* is the distance between the hinge points and the horizontal. It can be concluded that the intersection points of this mechanism are constrained to move along a horizontal line.

**Figure 8:**
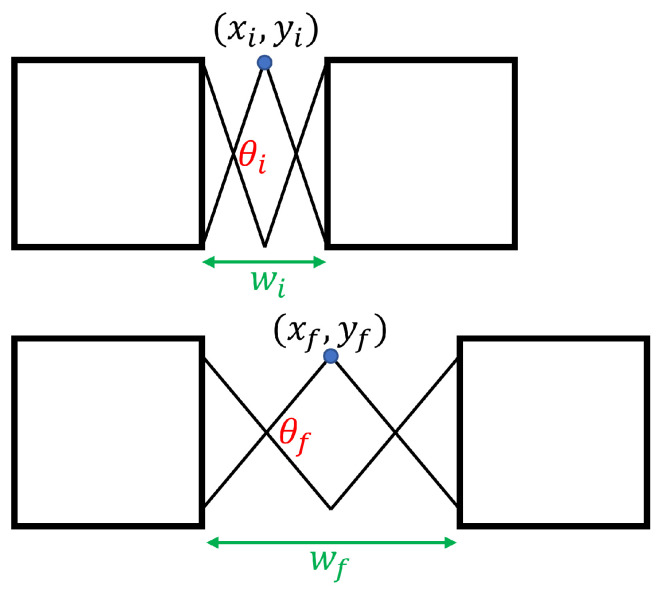
The movement of a single scissor-hinge unit. Each link has length *l*.

Given this relationship, it must be determined how a single scissor-hinge unit moves during deployment. Figure 8 shows the movement path of the interior point of a scissor-hinge unit during its operation.

Again, by geometry, the movement of the interior point can be determined. It can also be related to the distance between the hinges on either side:

(3) $$x_{f}-x_{i}=l(\cos\left(\frac{\theta_{f}}{2}\right)-\cos\left(\frac{\theta_{i}}{2}\right))=\frac{1}{2}\left(w_{f}-w_{i}\right)$$

(4) $$y_{f}-y_{i}=l\left(\sin\left(\frac{\theta_{f}}{2}\right)-\sin\left(\frac{\theta_{i}}{2}\right)\right)$$

**Figure 9:**
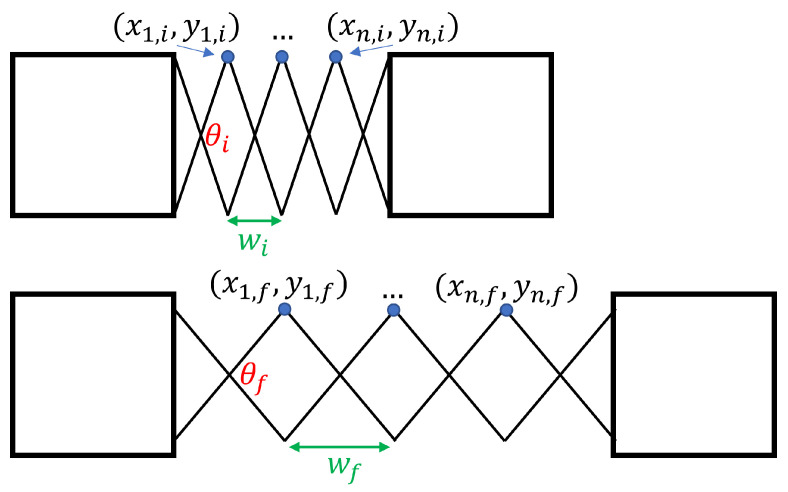
The movement of an entire scissor-hinge assembly.

The above may now be extended to determine the movement of an entire mechanism during its deployment, as shown in Figure 9.

The described technique relies on an assembly of four scissor-hinge mechanisms connected by four square corner blocks, as shown in Figure 3.

The movement paths of each perimeter node of the network during deployment can now be determined. Let the interior hinge positions be represented as four sets:

1. The topmost *j* hinges $\left(x_{t,j},y_{t,j}\right)$, where *j* runs from 1 to *m*.
2. The bottommost *j* hinges $\left(x_{b,j},y_{b,j}\right)$, where *j* runs from 1 to *m*.
3. The leftmost *k* hinges $\left(x_{l,k},y_{l,k}\right)$, where *k* runs from 1 to *n*.
4. The rightmost *k* hinges $\left(x_{r,k},y_{r,k}\right)$, where *k* runs from 1 to *n*.

Let the angles associated with the scissor-hinge mechanisms be $\theta_{h}$ and $\theta_{v}$ for the horizontal- and vertical-running mechanisms, respectively. Let *a* be the side length of one corner block, *w* be the horizontal hinge-to-hinge distance, *h* be the vertical hinge-to-hinge distance, $l_{v}$ be the half-length of the vertical hinge link, and $l_{h}$ be the half-length of the horizontal hinge link. Then, a set of kinematics equations can be derived as follows:

(5) $$x_{b,j}=x_{t,j}=a+w\left(j-\frac{1}{2}\right)$$

(6) $$x_{l,k}=\frac{a}{2}+l_{v}\sin\frac{\theta_{v}}{2}$$

(7) $$y_{b,j}=\frac{a}{2}+l_{h}\sin\frac{\theta_{h}}{2}$$

(8) $$x_{r,k}=\frac{3a}{2}-l_{v}\sin\frac{\theta_{v}}{2}$$

(9) $$y_{t,j}=\frac{3a}{2}-l_{h}\sin\frac{\theta_{h}}{2}$$

(10) $$y_{l,k}=y_{r,k}=a+h\left(k-\frac{1}{2}\right)$$

This set of kinematics equations expresses the positions of each interior point of the scissor-hinge mechanisms in terms of the opening angles $\theta_{h}$ and $\theta_{w}$. However, these parameters are not directly controlled by the user. Rather, the mechanism is controlled as shown in Figure 4, i.e., by expanding the entire scissor-hinge mechanism along its length. As such, the variables *W* and *H* are introduced to represent the length of each scissor-hinge mechanism: *W* corresponding to the length of the mechanisms along the *x* direction and *H* corresponding to the length of the mechanisms along the *y* direction. Now these control parameters can be related to the positions of each interior point:

(11) $$x_{b,j}=x_{t,j}=a+\frac{W}{m}\left(j-\frac{1}{2}\right)$$

(12) $$x_{l,k}=\frac{a}{2}+l_{v}\sqrt{1-{\left(\frac{H}{2nl_{v}}\right)}^{2}}$$

(13) $$y_{b,j}=\frac{a}{2}+l_{h}\sqrt{1-{\left(\frac{W}{2ml_{h}}\right)}^{2}}$$

(14) $$x_{r,k}=\frac{3a}{2}-l_{v}\sqrt{1-{\left(\frac{H}{2nl_{v}}\right)}^{2}}$$

(15) $$y_{t,j}=\frac{3a}{2}-l_{h}\sqrt{1-{\left(\frac{W}{2ml_{h}}\right)}^{2}}$$

(16) $$y_{l,k}=y_{r,k}=a+\frac{H}{n}\left(k-\frac{1}{2}\right)$$

By controlling the rate of change of *W* and *H*, the listed positions can be changed at a controlled velocity.

(17) $${\dot{x}}_{b,j}={\dot{x}}_{t,j}=\frac{\dot{W}}{m}\left(j-\frac{1}{2}\right)$$

(18) $${\dot{x}}_{l,k}=-\frac{H\dot{H}}{4n^{2}l_{v}\sqrt{1-{\left(\frac{H}{2nl_{v}}\right)}^{2}}}$$

(19) $${\dot{y}}_{b,j}=-\frac{W\dot{W}}{4m^{2}l_{h}\sqrt{1-{\left(\frac{W}{2ml_{h}}\right)}^{2}}}$$

(20) $${\dot{x}}_{r,k}=-\frac{H\dot{H}}{4n^{2}l_{v}\sqrt{1-{\left(\frac{H}{2nl_{v}}\right)}^{2}}}$$

(21) $${\dot{y}}_{y,j}=-\frac{W\dot{W}}{4m^{2}l_{h}\sqrt{1-{\left(\frac{W}{2ml_{h}}\right)}^{2}}}$$

(22) $${\dot{y}}_{l,k}={\dot{y}}_{r,k}=\frac{\dot{H}}{n}\left(k-\frac{1}{2}\right)$$

Thus can the kinematics of the tool be described using *W*, *H*, and the design variables of the tool. At this stage, the movement resolution of the tool may be calculated.

Regarding movement resolution, of concern is the minimum amount that the assembly can move in one control signal. The minimum amount that one interconnect can be stretched must be determined. If this value is too large, it introduces the risk of damaging the interconnect by applying tension too quickly. Recall that

(23) $${\dot{x}}_{b,j}={\dot{x}}_{t,j}=\frac{\dot{W}}{m}\left(j-\frac{1}{2}\right)$$

The maximum of the index *j* is *m*, which is 8. Let δx be the smallest possible movement of the hinge. This is related to δW, which is the smallest possible movement of the belt drive. The pitch of the belt drive used in the tool is 2 mm; this is the δW for the tool, as this displacement occurs during each “tick” of the stepper motor, which is the minimum bit of control available. Of interest is how much two adjacent nodes move relative to each other during one such bit, as this corresponds to the minimum amount that one interconnect can be stretched. Let this distance be δl, which can be calculated as

(24) $$\delta l=\delta x_{j}-\delta x_{j-1}=\frac{\delta W}{m}\left(j-\frac{1}{2}\right)-\frac{\delta W}{m}\left((j-1\right)-\frac{1}{2})=\frac{\delta W}{m}=\frac{2\;\mathrm{mm}}{8}=0.25\;\mathrm{mm}$$

The maximum allowed value in this case is unknown, but a value on the order of the one calculated above is likely to be compatible with the stretching process. The value can be reduced further by decreasing the pitch value of the timing belt and gear assembly.

#### 3.2.3. Compatibility

Another crucial design requirement of the stretch tool is compatibility. When the tool is stretched, each perimeter node is moved from some initial position (its manufactured footprint) to some final position (its deployed footprint). The scissor-hinge mechanisms must be designed such that they include both of these positions on their movement paths. The network can also be designed to help accommodate the compatibility requirement.

**Figure 10:**
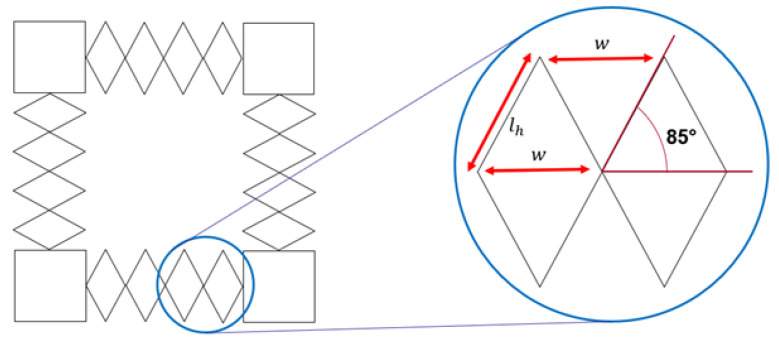
The initial configuration of the scissor-hinge mechanism.

First, it must be established that the tool has sufficient range to expand the network to the appropriate size. To maximize expansion, the tool should be nearly fully contracted in its initial position. In this position, the links in the scissor-hinge mechanism will make some angle to the horizontal. Care must be taken to leave the mechanism partially open in its initial configuration, or else the mechanism becomes singular and exhibits unpredictable opening behavior. In practice, an angle of $85^{{}^{\circ}}$ is low enough to ensure predictable opening of the scissor-hinge mechanism. This configuration is shown in Figure 10.

In the tool’s initial configuration, assuming this $85^{{}^{\circ}}$ value, then $w=2l_{h}\cos85^{{}^{\circ}}=0.174l_{h}$. With this initial configuration, the following stretch factors can be produced.

The scissor-hinge mechanism can thus stretch by a factor of greater than 10 if it starts $5^{{}^{\circ}}$ from completely closed and ends $5^{{}^{\circ}}$ from completely open, making it appropriate for stretching the SSN, which has a stretch ratio of 10 on a side. The $5^{{}^{\circ}}$ assumption informs the requirement for link length, as the link length is also constrained by the distance between edge pads.

**Figure 11:**
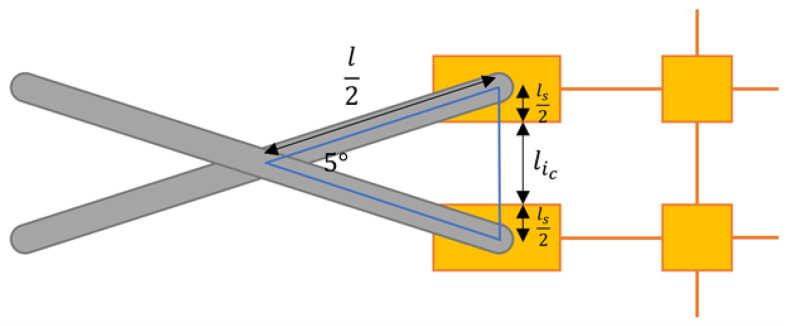
Geometry of the closed scissor-hinge unit.

To select the appropriate link length, the geometry of the tool is considered in the closed configuration, as in Figure 11. Let the length of a scissor hinge link equal *l*, the length of a sensor pad on one side be $l_{s}$, and the length of a closed interconnect be $l_{i_{c}}$.

After substituting in the values, the result is

(25) $$\frac{1}{2}=\frac{\left(l_{s}+l_{i_{c}}\right)\sin\left(87.5^{{}^{\circ}}\right)}{\sin(5^{{}^{\circ}})}=22.9\left(l_{s}+l_{i_{c}}\right)$$

Therefore, the length of each link should be roughly 23 times the distance between the centers of the edge pads. However, the kinematics of the scissor-hinge assembly have seemingly now been fully defined. In order to achieve compatibility, another design parameter is required. Here, an edge interconnect is defined as an interconnect that connects any node to an edge node. These interconnects are different in shape to the remaining interconnects and must be designed separately. Specifically, they must be designed to have a closed length in the initial tool configuration and an open length in the final tool configuration.

**Figure 12:**
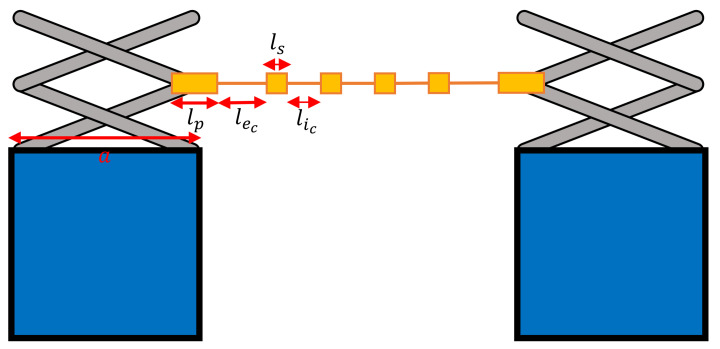
Geometry of the closed scissor-hinge tool.

These lengths can be determined from the tool geometry. Consider a line with length d intersecting two interior hinge points on opposite sides of the tool, as in Figure 12. Let the length of the edge pad equal $l_{p}$, the length of the closed edge interconnect be $l_{e_{c}}$, and the length of one side of the corner block be *a*.

Two expressions can be constructed for *d*; one in terms of the scissor hinge length and one in terms of the sensor network geometry. Assuming *n* number of sensors in a row and setting the two expressions to be equal,

(26) $$l_{p}+nl_{s}+\left(n-1\right)l_{i_{c}}+2l_{e_{c}}=n\left(l_{i_{c}}+l_{s}\right)+2(\frac{a}{2}-22.9\left(l_{s}+l_{i_{c}}\cos\left(2.5^{{}^{\circ}}\right))\right)$$

(27) $$l_{e_{c}}=\frac{a}{2}-22.4l_{i_{c}}-\frac{l_{p}}{2}-22.9l_{s}$$

In this case, the open interconnect lengths can be considered, as well as the fact that the interior hinges retract relative to the corner blocks. It can be formulated that

(28) $$l_{p}+nl_{s}+\left(n-1\right)l_{i_{o}}+2l_{e_{o}}=n\left(l_{i_{o}}+l_{s}\right)+2(\frac{a}{2}-22.9(l_{s}+l_{i_{o}}\sin\left(2.5^{{}^{\circ}}\right))),$$

resulting in

(29) $$l_{e,o}\approx \frac{a}{2}-\frac{l_{i_{o}}}{2}-\frac{l_{p}}{2}-l_{s}$$

This calculation assumes that the stretch tool is opened from $5^{{}^{\circ}}$ from completely closed to $5^{{}^{\circ}}$ from completely open. In practice, the tool may not be required to open completely, and the open interconnect length should be adjusted accordingly.

Thus, to ensure compatibility, the link length, open and closed edge node length, and corner block length must be appropriately selected.

### 3.3. Stretch Tool Prototype

In this section, the general procedure in Section 3.2 is used to design a prototype that is capable of expanding a network with a specific configuration.

#### 3.3.1. Design Inputs

The stretch tool is designed to stretch a network with equally spaced edge nodes. In this testing, the configuration happens to be uniform, but the proposed principle can be extended to networks with non-uniform interconnect lengths and stiffnesses.

For the validation tests, a network was selected with eight edge nodes on a side with 3.4 mm spacing between each edge node. Each node itself has a width of 2.4 mm, thus the link length can be selected using Equation (25), which specifies that a link length of 90 mm is appropriate. These links were waterjet-cut out of a 1 mm thick aluminum sheet, then assembled using M1 nuts and screws to complete the scissor-hinge mechanisms.

A gear with the appropriate pitch must be selected. As specified in Equation (24), a movement bit of 0.25 mm/s is appropriate for this design, which means that the gear pitch should be 2 mm. By varying the stepper motor speed, the tool can be opened at a different rate; in the validation experiment, a maximum speed of 8 mm/s, or 2 ticks/s, was selected. The length of the belt must also be specified; it must span the width of the tool in its closed configuration, and should thus be selected to be 110 cm. This gear pitch was available commercially, and a larger belt was cut to length for the experiment. A hinged spring was used to tighten the belt and remove slack.

Finally, the open and closed edge node lengths as well as the corner block lengths must be selected. In this case, the selected network configuration had a closed edge node length of 9 mm, which was used to select a corner block size of 110 mm. This corner block was additively manufactured using a Form2 printer, and roller bearings were press-fit into the bottom of the blocks to allow them to roll over the work surface. When opened, the edge node length expanded to as much as 49 mm, which accommodates this design. Because the tested network was not designed to expand by an areal factor of 100, the final open angle of the scissor-hinge mechanisms was $56^{{}^{\circ}}$.

#### 3.3.2. Limitations

The method of approach detailed in Section 3.3.1 has some limitations. For instance, this tool is specified to stretch an 8×8 network. In order to stretch a smaller network, or a network with a different aspect ratio, the scissor-hinge mechanisms must be replaced with other mechanisms with the appropriate number of interior hinge points. While swapping out these mechanisms is relatively simple, having multiple scissor-hinge mechanisms on hand is a limitation of this design.

Second, the tool assumes that all edge nodes are equidistantly spaced. This configuration is appropriate for a large number of island-and-serpentine networks that are conceivably deployed, including ones with non-uniform interior nodes. However, work carried out in [49] shows that there is some design flexibility to be gained by using non-uniform edge node configurations for which this tool is not appropriate.

Finally, the tool is only appropriate for stretching networks up to a factor of 11.4 on each side, as detailed in Table 4. At the time of this writing, this is largely appropriate for island-and-serpentine networks currently in use. For expansions with a factor greater than 11.4, however, a different method of approach is required for deployment.

## 4. Analysis of Results

After the fabrication of the SSN and stretch tool assembly, test expansions must be performed to characterize the tool’s appropriateness for the expansion process.

### 4.1. Relevant Metrics

The purpose of the tool is to solve existing problems with manual deployment of stretchable sensor networks; hence, it is necessary to evaluate to what degree these problems are solved by evaluating the tool on a series of metrics. As such, the relevant problems and their corresponding metrics are addressed as follows.

The first metric of interest is the total time taken for the process to proceed; with manual methods, this can take several hours. Simply stated, the shortest possible deployment time is desired. However the goal of this tool is to decrease the time taken for the process to proceed significantly, and a goal of a two orders of magnitude reduction in process time (i.e., a process that takes minutes rather than multiple hours) is proposed as a representative benchmark.

The second metric of interest centers on the safety of the process. Here, the safety metric is defined as the number of interconnects which break during the expansion process; as outlined earlier in this paper, manual methods introduce processes that expose individual interconnects to risk of tangling or breaking. Even a single snapped interconnect displaces the remaining interconnects and damages the signal paths in the network. Thus, to be considered successful, the process must proceed without a single interconnect snapping or becoming tangled.

The final metric concerns the uniformity of the deployed network. The tool must deploy sensor nodes in uniform locations on the target surface. In this study, the target deployment configuration is a uniform 8×8 square grid where each node is spaced nominally 39 mm apart, but the metric of interest is whether the nodes occupy a uniform grid configuration rather than whether they occupy a grid with exactly 39 mm spacing. As a metric for uniformity, the average positional error of each node from a uniform grid of best fit is used. The sensors used in the network should be positioned within approximately 10 mm of their locations on a uniform grid on average for the tool to be considered successful.

To characterize uniformity, the recorded data are compared to the uniform grid that best fits the data. To accomplish this, this best fit grid must be determined. This grid has three parameters: the grid origin relative to the image data, the grid rotation relative to the image data, and the grid spacing; these parameters must be selected to minimize the positional error in the grid.

In this study, the grid parameters were determined by creating the an error function in Python, then using the Nelder–Mead simplex algorithm to minimize this error function in the grid parameters.

Specifically, this process proceeds as follows:

1. The raw test data are read into an array of x- and y-positions.
2. A uniform 8×8 grid is generated with some uniform spacing, origin (x,y), and some angle of rotation θ.
3. An error function is constructed. This is $\sqrt{{\left(x_{raw}-x_{grid}\right)}^{2}+{\left(y_{raw}-y_{grid}\right)}^{2}}$, where “raw” subscripts correspond to experimental data and “grid” subscripts correspond to the proposed best fit grid.
4. The Python library scipy.optimize.minimize is used to find the best fit grid subject to the above error function by selecting the appropriate origin, spacing, and angle of rotation.

Once the best fit grid is determined, the mean and standard deviation of the Euclidean distance from each node to its best fit grid counterpart is reported.

The following procedure was used to perform test expansions:

1. The network was loaded into the tool with an alignment plate. Each edge node was inspected to ensure a proper mechanical connection with the scissor-hinge structures.
2. Using the optical encoder, the tool was manually ramped up to a constant expansion rate.
3. The expansion process was allowed to proceed until the network was completely stretched.
4. Using the optical encoder, the expansion process was halted.

A stopwatch was used to measure the total expansion time, and interconnects were manually inspected for damage after each expansion process.

To measure sensor locations, an overhead photo of the final network configuration was taken using a level tool to prevent perspective distortion. Using image processing software (ImageJ), the relative locations of each sensor on the build plate were recorded. ImageJ is a public domain software developed at the National Institutes of Health and the Laboratory for Optical and Computational Instrumentation (LOCI, University of Wisconsin, Madison, WI, USA).

**Figure 13:**
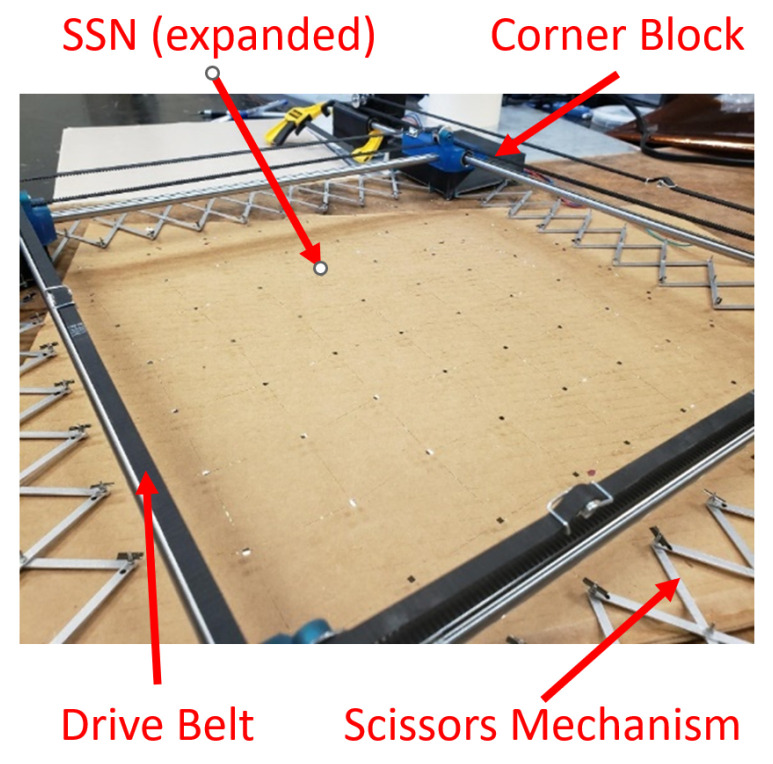
Post-stretch tool configuration.

Two square networks were manufactured and expanded with the tool to their design size. In each case, the tool was manually controlled with the optical encoder to obtain the desired speed. The manufactured networks are designed to cover an area with a uniform grid of nodes spaced nominally 39 mm apart on a side. The total active area is thus nominally 273 mm on a side, covering $745\;{\mathrm{cm}}^{2}$. An example of a stretched network is pictured in Figure 13.

### 4.2. Example Deployment

**Figure 14:**
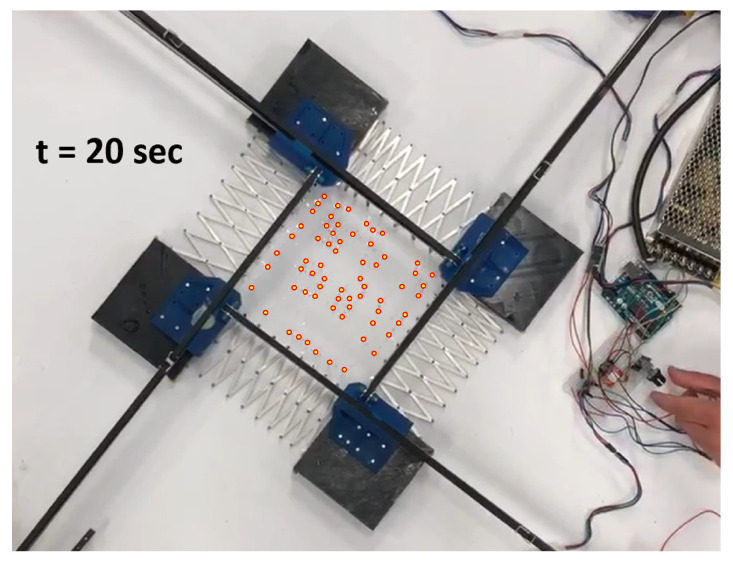
Stretch at 20 s. Red-outlined circles indicate sensor node locations.

**Figure 15:**
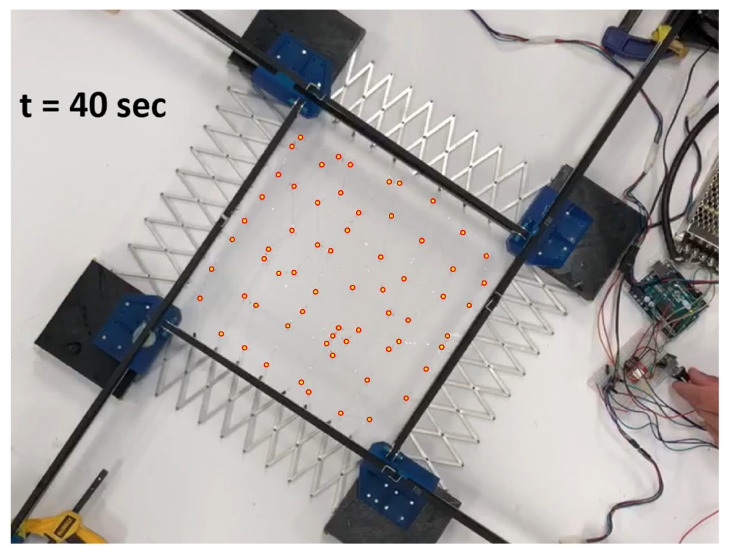
Stretch at 40 s. Red-outlined circles indicate sensor node locations.

**Figure 16:**
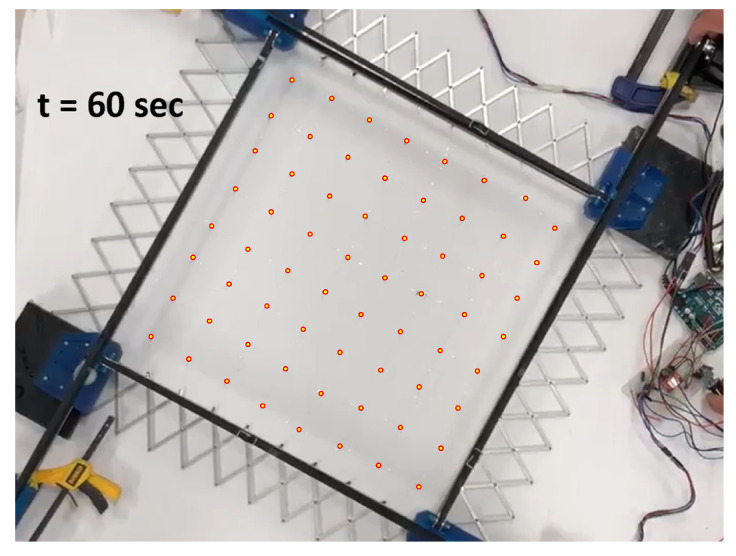
Stretch at 60 s. Red-outlined circles indicate sensor node locations.

During expansion, the network initially covers its manufacturing footprint in the fully contracted tool. As the network is expanded, sacrifice bridges begin to open. There is stochasticity to this process; multiple bridges should break simultaneously when viewed from a quasistatic standpoint, but in fact variations in the strength of these bridges result in a highly random breaking order. An example of this phenomenon can be seen in Figure 14, Figure 15 and Figure 16.

### 4.3. Speed

Once loaded into the tool, the opening speed of the tool (i.e., the rate of change of *W* and *H*) can be manipulated from 2 mm/s to 20 mm/s.

#### 4.3.1. Run 1

During the first network expansion, very conservative speed control was employed. During expansion, the opening speed was ramped up by 2 mm/s${}^{2}$ from 0 to 4 mm/s. The opening speed was left at 4 mm/s for the duration of the opening, then ramped down by 2 mm/s${}^{2}$ for the last two seconds of deployment. The total process time was 61 s.

#### 4.3.2. Run 2

During the second network expansion, a less conservative approach was used. During expansion, the opening speed was ramped up by 2 mm/s${}^{2}$ from 0 to 8 mm/s. The opening speed was left at 8 mm/s for the duration of the opening, then ramped down by 2 mm/s${}^{2}$ for the last four seconds of deployment. The total process time was 33 s.

#### 4.3.3. Evaluation

It was shown that the tool process time was as small as 33 s without sacrificing the safety of the interconnects; the tool meets this metric for success.

### 4.4. Uniformity

During deployment, the edge nodes of the network are positionally controlled. Theoretically, the interconnects, which are all of identical stiffness, should cause the sensor nodes to remain uniformly spaced once they have reached static equilibrium.

#### 4.4.1. Run 1

**Figure 17:**
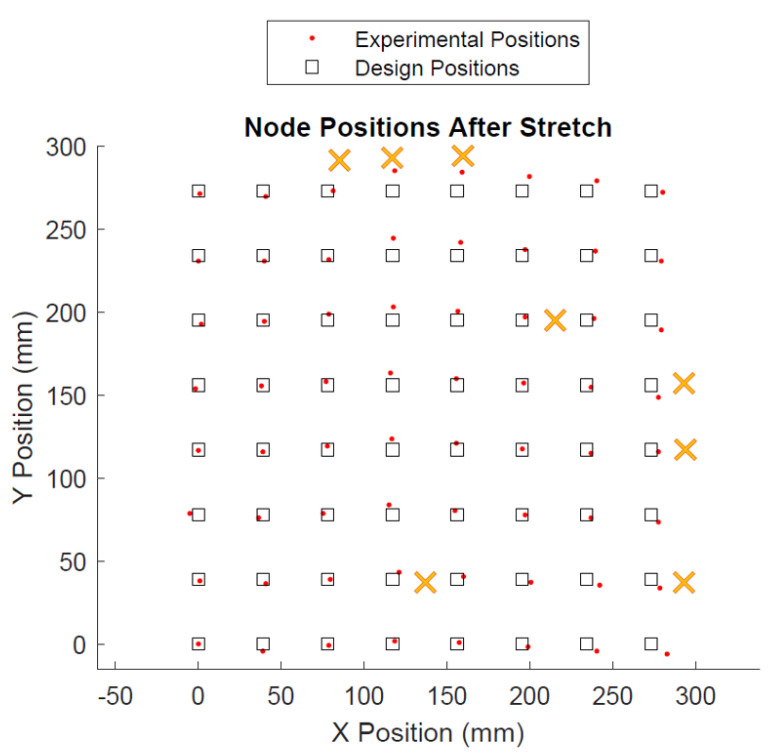
Node positions after Run 1. Xs indicate locations of unopened sacrifice bridges.

The positions of the sensor nodes after the first expansion are shown in Figure 17.

**Figure 18:**
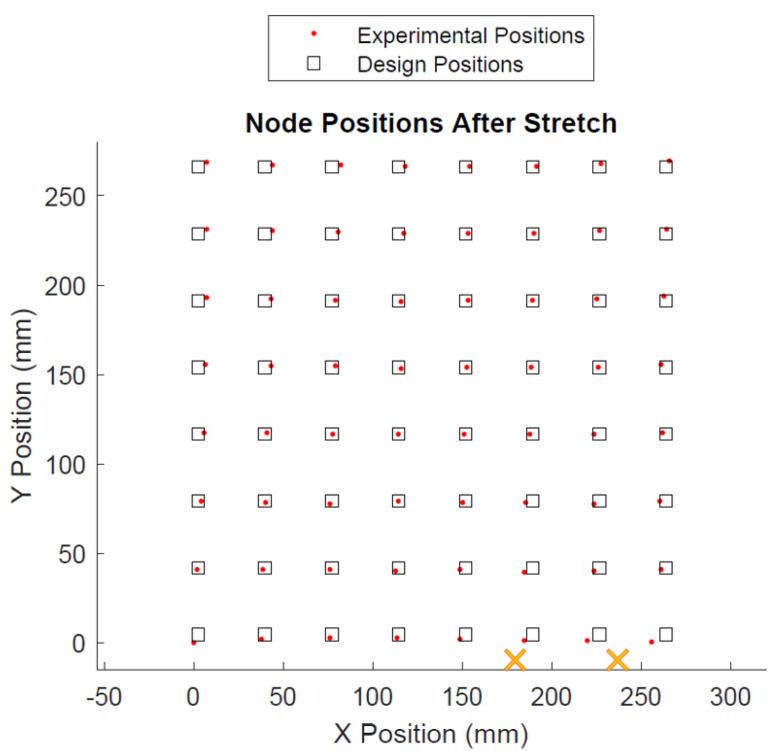
Node positions after Run 2. Xs indicate locations of unopened sacrifice bridges.

In Figure 17 and Figure 18, the desired position of the network nodes are displayed as boxes, and the actual deployed positions as red dots. The network is designed to relax to a uniform grid configuration with nodes spaced nominally 39 mm apart. Based on Euclidean distance, the mean positional error was 4.1 mm ± 2.2 mm, or 1.4% of one side of the square area. As defined in the metrics section, this is appropriate for a successful deployment tool. Note, however, that there are outliers. In particular, the two center nodes at the top of the formation are furthest from their positions due to unopened sacrifice bridges. Individual positional data for each node can be found in Appendix A.

#### 4.4.2. Run 2

The positions of the sensor nodes after the second expansion are shown in Figure 18.

Based on Euclidean distance, the mean positional error was 2.8 mm ± 1.8 mm, or 1.1% of one side of the square area. Individual positional data for each node can be found in Appendix A.

#### 4.4.3. Evaluation

As defined in the metrics section, both runs represent a successful deployment, but further analysis is warranted to address outliers.

In both cases, the main source of error can be attributed to unopened sacrifice bridges. As shown in Figure 2, sacrifice bridges comprise several bridge structures that keep the unopened interconnect relatively stationary during transportation and loading into the tool. Unlike the serpentines, which open via unfolding, the sacrifice bridges are made of polyimide and break rather than unfolding. As a result, if only some bridge structures snap, the interconnect can become only partially unfolded, effectively remaining stiffer than the neighboring interconnects. Because sensors relax to a static equilibrium that is based on the interconnect tensions on each side of the sensor, this causes the sensors to become biased toward the positions of these partially opened bridges. Likewise, this bias is passed onto the neighboring sensors, displacing them as well. Figure 17 and Figure 18 show the location of partially opened bridges.

It was observed that fewer sacrifice bridges were left unopened in Run 2, the run where less conservative speed control was used. In both cases, no interconnects were observed to break or become tangled, leaving the network intact. As summarized above, some interconnects remained partially unopened.

### 4.5. Safety

#### Evaluation

In both cases, no interconnect was observed to snap or become tangled, and the tool performs appropriately on this metric. As noted previously, however, not all sacrifice bridges opened during deployment.

## 5. Integration

After deployment, the network can be integrated into a composite structure in situ. At SACL, the process for integrating one such structure into a composite panel is summarized as follows:

1. A flexible printed circuit board with pads to accommodate the edge nodes is fixed to the plate with an adhesive. Each terminal on the board connects each pad to a pin on a D-Sub connector.
2. The stretch tool is used to stretch the network in situ, such that the edge nodes rest above the pads on the printed circuit board.
3. Each edge node is removed from the tool using tweezers, then attached to the circuit board using silver paste.
4. The entire assembly is covered with a fiberglass veil.
5. This assembly is vacuum bagged and cured in an oven.

**Figure 19:**
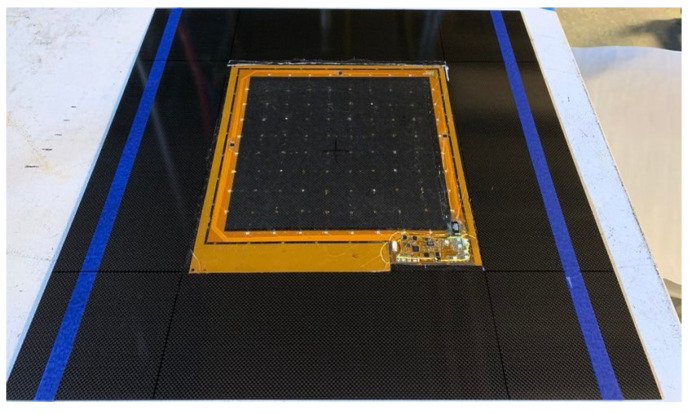
An SSN integrated with a composite panel.

Figure 19 shows an example of a completed panel with strain gauges, RTDs, and PZT sensors [50].

## 6. Conclusions and Future Work

The method implemented in this paper improves significantly on the manual deployment methods for island-and-serpentine networks. Specifically, the test networks were stretched in 61 s or less, as opposed to in several hours, as in existing technologies; this was accomplished with no damage to the network and with a minimum of human intervention (i.e., loading the tool). The resulting stretched network had sufficient uniformity for use in structural health monitoring applications.

Despite these successes, sensors sometimes become displaced from their design locations by unopened sacrifice bridges, especially those bridges at the edges of the network. These bridges required more force to open than was anticipated. During the manufacturing process, these bridges may be produced with some variance in their failure strength. This variance should be characterized to determine whether it affects this behavior; if the variance in bridge strength is too great, then the strongest bridges will likely remain open at the conclusion of the stretch process. It was noted that the second, more rapid expansion resulted in fewer unopened sacrifice bridges; it is possible that this increase in speed introduced dynamic loading, causing bridges to open more effectively.

## Figures and Tables

**Table 1:** Summary of the expandable sensor network literature (sensor types and applications).

Author, Date, Reference	Sensor Type	Application
Lanzara et al., 2010, [36]	PZT, RTD, Strain	Structural Health Monitoring
Xu et al., 2012, [40]	None	Structural Study
Zhang et al., 2013, [38]	None	Structural Study
Fan et al., 2013, [41]	None	Electrode, Antenna
Jang et al., 2014, [33]	None	Structural Study
Gutbrod et al., 2014, [37]	pH, LED	Medical
Hua et al., 2018, [13]	Various	Various
Wang et al., 2019, [3]	PZT	Structural Health Monitoring

**Table 2:** Summary of the expandable sensor network literature (geometries and deployment methods).

Author, Date, Reference	Geometry	Deployment
Lanzara et al., 2010, [36]	“S” interconnect	Manual
Xu et al., 2012, [40]	Self-Similar	Unspecified
Zhang et al., 2013, [38]	“S” interconnect	Specialized Fixture
Fan et al., 2013, [41]	Fractal	Not Stretched
Jang et al., 2014, [33]	Fractal	Unspecified
Gutbrod et al., 2014, [37]	“S” interconnect	Not Stretched
Hua et al., 2018, [13]	“S” interconnect	Unspecified
Wang et al., 2019, [3]	Self-Similar	Unspecified

**Table 3:** Design inputs and outputs.

Input (Parameter)	Output (Design Requirement)
Belt pitch, motor speed	Movement resolution, velocity
Link length, edge interconnect length, corner block length	Compatibility, boundary conditions, total expanded footprint

**Table 4:** Stretch factors for selected opening angles.

Initial Opening Angle (Degrees)	*w*	Ratio to Initial *w*
85	0.174$l_{h}$	1
75	0.517$l_{h}$	2.97
65	0.845$l_{h}$	4.86
45	1.41$l_{h}$	8.10
35	1.64$l_{h}$	9.43
25	1.81$l_{h}$	10.4
15	1.93$l_{h}$	11.1
5	1.99$l_{h}$	11.4

## Data Availability

Not applicable.

## Appendix A. Raw Data

In this section, the positional data used in Section 4 is listed. The “Target” values represent the values (in the X and Y directions, as reflected in Figure 17 and Figure 18) that the network nodes would occupy on the grid of best fit, and which they would occupy if the stretch was performed with complete uniformity. The “Actual” values reflect the actual positions of the nodes in the stretched network. The “Error” values reflect the positional error between the target and actual values, which was obtained by calculating the distance between each pair of points in the XY-plane using the Pythagorean theorem.

**Table A1:** Run 1 positional data (lower half of the network).

Target X (mm)	Target Y (mm)	Actual X (mm)	Actual Y (mm)	Error (mm)
0	0	0	0	0
39.5	0	38.885	−4.32	4.36
79	0	78.634	−0.864	0.93
118.5	0	118.382	1.728	1.73
158	0	157.267	0.864	1.13
197.5	0	198.744	−1.728	2.12
237	0	240.221	−4.32	5.38
276.5	0	282.562	−6.048	8.56
0	39.5	0.864	38.021	1.71
39.5	39.5	40.613	36.293	3.39
79	39.5	79.498	38.885	0.79
118.5	39.5	120.975	43.205	4.45
158	39.5	159.859	40.613	2.16
197.5	39.5	200.472	37.157	3.78
237	39.5	241.949	35.428	6.40
276.5	39.5	278.241	33.7	6.05
0	79	−5.184	78.634	5.19
39.5	79	36.292	76.041	4.36
79	79	75.177	78.634	3.84
118.5	79	114.926	83.818	5.99
158	79	154.675	80.362	3.59
197.5	79	197.016	77.769	1.32
237	79	236.764	76.041	2.96
276.5	79	277.377	73.449	5.61
0	118.5	0	116.654	1.84
39.5	118.5	38.885	115.79	2.77
79	118.5	77.769	119.246	1.43
118.5	118.5	116.654	123.567	5.39
158	118.5	155.539	120.975	3.49
197.5	118.5	195.287	117.518	2.42
237	118.5	236.764	114.926	3.58
276.5	118.5	277.377	115.79	2.84

**Table A2:** Run 1 positional data (upper half of the network).

Target X (mm)	Target Y (mm)	Actual X (mm)	Actual Y (mm)	Error (mm)
0	158	−1.728	153.811	4.53
39.5	158	38.021	155.539	2.87
79	158	76.905	158.131	2.09
118.5	158	115.79	163.316	5.96
158	158	155.539	159.859	3.08
197.5	158	196.151	157.267	1.53
237	158	236.764	154.675	3.33
276.5	158	277.377	148.626	9.41
0	197.5	1.728	192.695	5.10
39.5	197.5	39.749	194.423	3.08
79	197.5	78.634	198.744	1.29
118.5	197.5	117.518	203.064	5.64
158	197.5	156.403	200.472	3.37
197.5	197.5	197.016	197.016	0.68
237	197.5	238.492	196.152	2.01
276.5	197.5	279.105	189.239	8.66
0	237	0	230.716	6.28
39.5	237	39.749	230.716	6.28
79	237	78.634	231.58	5.43
118.5	237	117.518	244.541	7.60
158	237	158.131	241.949	4.95
197.5	237	197.016	237.628	0.79
237	237	239.357	236.764	2.36
276.5	237	279.105	230.716	6.80
0	276.5	0.864	271.328	5.24
39.5	276.5	40.613	269.6	6.98
79	276.5	81.226	273.057	4.09
118.5	276.5	118.382	285.154	8.65
158	276.5	158.995	284.29	7.85
197.5	276.5	199.608	281.698	5.60
237	276.5	240.221	279.105	4.14
276.5	276.5	279.969	272.193	5.53

**Table A3:** Run 2 positional data (lower half of the network).

Target X (mm)	Target Y (mm)	Actual X (mm)	Actual Y (mm)	Error (mm)
2.46	4.52	0.00	0.00	5.15
39.81	4.52	37.85	1.91	3.27
77.16	4.52	76.07	2.68	2.14
114.52	4.52	113.54	2.68	2.09
151.87	4.52	148.71	1.91	4.10
189.22	4.52	184.64	1.15	5.69
226.57	4.52	219.81	1.15	7.55
263.92	4.52	255.75	0.38	9.16
2.46	41.87	1.91	40.90	1.12
39.81	41.87	38.61	40.90	1.55
77.16	41.87	76.07	40.90	1.46
114.52	41.87	112.77	40.14	2.46
151.87	41.87	148.71	40.90	3.31
189.22	41.87	184.64	39.37	5.21
226.57	41.87	223.63	40.14	3.41
263.92	41.87	261.10	40.90	2.99
2.46	79.22	4.20	79.13	1.74
39.81	79.22	40.14	78.37	0.92
77.16	79.22	76.07	77.60	1.95
114.52	79.22	114.30	79.13	0.23
151.87	79.22	150.24	78.37	1.84
189.22	79.22	185.41	78.37	3.91
226.57	79.22	223.63	77.60	3.35
263.92	79.22	260.33	79.13	3.59
2.46	116.57	5.73	117.36	3.36
39.81	116.57	40.90	117.36	1.34
77.16	116.57	77.60	116.60	0.44
114.52	116.57	114.30	116.60	0.21
151.87	116.57	151.00	116.60	0.87
189.22	116.57	187.70	116.60	1.52
226.57	116.57	223.63	116.60	2.94
263.92	116.57	261.86	117.36	2.20

**Table A4:** Run 2 positional data (upper half of the network).

Target X (mm)	Target Y (mm)	Actual X (mm)	Actual Y (mm)	Error (mm)
2.46	153.93	6.50	155.59	4.36
39.81	153.93	43.20	154.82	3.50
77.16	153.93	79.13	154.82	2.16
114.52	153.93	115.83	153.29	1.46
151.87	153.93	152.53	154.06	0.68
189.22	153.93	188.46	154.06	0.77
226.57	153.93	225.93	154.06	0.66
263.92	153.93	261.10	155.59	3.28
2.46	191.28	7.26	193.05	5.12
39.81	191.28	43.20	192.29	3.53
77.16	191.28	79.13	191.52	1.98
114.52	191.28	115.83	190.76	1.41
151.87	191.28	153.29	191.52	1.45
189.22	191.28	189.23	191.52	0.25
226.57	191.28	225.16	192.29	1.73
263.92	191.28	262.63	193.81	2.85
2.46	228.63	7.26	231.28	5.48
39.81	228.63	43.96	230.51	4.56
77.16	228.63	80.66	229.75	3.67
114.52	228.63	117.36	228.99	2.87
151.87	228.63	153.29	228.99	1.47
189.22	228.63	189.99	228.99	0.85
226.57	228.63	226.69	230.51	1.89
263.92	228.63	264.15	231.28	2.66
2.46	265.98	7.26	268.74	5.54
39.81	265.98	43.96	267.21	4.33
77.16	265.98	82.19	267.21	5.17
114.52	265.98	118.12	266.45	3.64
151.87	265.98	154.06	266.45	2.24
189.22	265.98	191.52	266.45	2.35
226.57	265.98	227.46	267.98	2.19
263.92	265.98	265.68	269.51	3.94
